# Vaccine Immunotherapy for Celiac Disease

**DOI:** 10.3389/fmed.2018.00187

**Published:** 2018-06-26

**Authors:** Antonio Di Sabatino, Marco V. Lenti, Gino R. Corazza, Carmen Gianfrani

**Affiliations:** ^1^First Department of Internal Medicine, San Matteo Hospital Foundation, University of Pavia, Pavia, Italy; ^2^Institute of Protein Biochemistry-National Research Council, Naples, Italy

**Keywords:** dendritic cell, epitope-specific immunotherapy, gliadin peptide, gluten-free diet, Nexvax2

## Abstract

Autoimmune and allergic disorders are highly prevalent conditions in which an altered or abnormal immune response is mounted against self- or environmental antigens, respectively. Antigen-based immunotherapy is a therapeutic option aimed at restoring the specific immune tolerance toward pathogenic antigens while leaving the rest of the immune system unaffected. This strategy proved efficacy especially in allergic diseases, including asthma, allergic rhinitis, and food allergies, but still has shortcomings for the treatment of autoimmune diseases. However, there are no available therapies, currently, in clinical practice for restoring the physiological tolerance that is typically lost in autoimmune diseases. In celiac disease, which is a common immune-mediated enteropathy triggered by the ingestion of gluten in genetically susceptible individuals, antigen-based immunotherapy could be a feasible option thanks to our deep understanding of the pathogenic mechanisms underpinning this condition. In fact, the immunodominant gluten epitopes are well-characterized and are recognized by pathogenic CD4^+^ T-cells that could be desensitized with immunotherapy. Moreover, the intestinal damage occurring in celiac disease (i.e., villous atrophy) is reversible upon gluten withdrawal. Only recently the results of a phase I trial of an intradermal, adjuvant-free, formulation of three specific gluten peptides (Nexvax2) showed a good safety profile, albeit its efficacy still needs to be demonstrated. More results are awaited, as they may radically change patients' quality of life that is constrained by the lifelong gluten-free diet and by the potential onset of life-threatening complications.

## Introduction

Autoimmune disorders comprise a broad range of human medical conditions that are the result of an altered, or abnormal, immune response mounted by the adaptive immune system and directed against self-antigens (1). Approximately 80 different autoimmune disorders have been identified, with an overall estimated prevalence of around 4%, even if this picture may widely differ depending on the specific disease, different age groups, and geographic areas (2). Among these, celiac disease (CD), that affects up to 1 in 100 individuals, embodies a unique pathogenic model in which the inflammatory and apoptotic cascade leading to the intestinal damage (i.e., villous atrophy) only happens in the presence of an environmental trigger (i.e., gluten) in genetically susceptible individuals (i.e., human leukocyte antigen [HLA] DQ2^+^ or DQ8^+^) (3). The only available therapy for CD is a lifelong and strict gluten-free diet (GFD). However, the better understanding of the pathological mechanisms underlying CD has paved the way to alternative therapies, including those able to restore a physiological immune response to gluten ingestion (4–6). We herein critically discuss the most recent advances and clinical potential of antigen-based immunotherapy (AIT) in CD.

## Antigen-based immunotherapy and its application in autoimmunity and allergy

The study of new possible effective and safe treatments for autoimmune and allergic disorders is an absolute priority, especially if we consider that these conditions are often chronic, disabling, and deeply affecting the quality of life. Disappointingly, despite a dramatic improvement of the medical treatments over the last decades, most of the available therapies are either non-specific (e.g., corticosteroids, immunomodulators, etc.) or are burdened by potentially severe side effects (e.g., anti-tumor necrosis factor α agents, anti-CD20 antibody, anti-integrins) (7–9). An intriguing alternative therapeutic strategy would be to restore the physiological T-cell tolerance toward specific antigens, thus preventing the subsequent inflammatory cascade, without altering the whole immune system at the same time. This represents the rationale for the use of AIT, that is the enteral or parenteral administration of immunodominant antigens or epitopes. Indeed, the first and main obstacle to be overcome is the detection of the immunodominant disease-trigger epitopes, and this information is still missing for many autoimmune conditions. Another issue that must be taken into account is that there might be more than one immunogenic epitope to be tolerated for obtaining a clinically relevant result. For example, many different autoantigens play a major pathogenic role in type I diabetes, multiple sclerosis, and systemic lupus erythematosus (10–12). Instead, this is not the case of allergic diseases in which the provocative antigen is usually known and well-classified.

Despite a few promising results of early phases clinical trials in humans, the use of AIT for autoimmune diseases has not yet been brought into practice (13). This could be partially explained by the fact that by the time an autoimmune disease is diagnosed, tissue damage may have already occurred or become irreversible, as in the case of type I diabetes. In fact, insulin-based immunotherapy (either oral or intranasal) failed to demonstrate either a curative or preventive efficacy in this disease in at least three different trials (14–16). Other trials using GAD65-based immunotherapy are ongoing, but once again, the results accumulated so far failed to show a significant efficacy in preventing the onset of type I diabetes or in restoring pancreatic islet β-cells function (17, 18). Combination AITs could be a feasible option and a future research area, but more evidence is awaited (19). Multiple sclerosis is another complex immune-mediated disease with multiple identified autoantigens in which neurological lesions are unlikely to be completely healed after their onset. Nonetheless, preliminary results of an AIT with skin patches containing a mixture of three different myelin peptides are encouraging, showing a radiological and clinical reduction of multiple sclerosis activity during the 1-year treatment (20). Finally, only recently the preliminary results of a phase I trial with a peptide-based, epitope-specific immunotherapy (Nexvax2) for CD have been published, and these will be discussed later in a dedicated section (21, 22).

Differently to autoimmune diseases, AIT for allergic diseases is widely available and routinely used in clinical practice (23, 24). Antigen sensitization is a multistep process that involves different mechanisms, both on a short (i.e., mast cell and basophil desensitization) and a long-term (i.e., the induction of antigen-specific regulatory T-cell populations which in turn suppress antigen responding effector T-cells) (24). Interestingly, AIT for allergic diseases also influence the response of regulatory B-cell inducing them to produce a higher amount of the anti-inflammatory cytokine interleukin (IL)-10 (25). Multiple systematic reviews and meta-analyses showed a significant efficacy of AIT, which usually comes in sublingual or subcutaneous formulations, in relieving or preventing symptoms in patients suffering from asthma, allergic rhinitis, and food allergies, with an overall good safety profile, but with an unavoidable potential risk of anaphylaxis that should always be borne in mind (26–28).

## Celiac disease and its immune pathogenesis

CD is an immune-mediated enteropathy characterized by a wide clinical spectrum, spacing from the total lack of any symptom to severe diarrhea and malabsorption, and burdened by irreversible or potentially life-threatening complications, including refractory CD, ulcerative jejunoileitis, and enteropathy-associated T-cell lymphoma (3). Two concurrent factors are necessary for the onset of CD, namely the ingestion of gluten (that contains gliadins and glutenins) and the presence of HLA-DQ2 or -DQ8 haplotypes (29). However, given that one fourth of the general population carries these haplotypes and CD prevalence is approximately 1%, it follows that other additional factors are implicated. Genome-wide association studies have shown at least 41 non-HLA risk *loci*, most of them involving T-cell immune-response regulatory genes, such as IL-2 or IL-2, which are both involved in the onset and maintenance of mucosal damage in CD (30–32). Regarding environmental triggers, *rotavirus* infections during childhood may increase the risk of developing CD (33), but a more solid evidence is still awaited.

In CD intestinal permeability is impaired, due to the exaggerated enterocyte apoptosis (34), thus easing the translocation of gluten peptides across the intestinal epithelium (35). Paracellular translocation is a consequence of the increased release of zonulin after the binding of gluten peptides to the chemokine receptor CXCR3 (36). Gliadin peptides can also cross the epithelial barrier through transcytosis, that involves an interferon (IFN)-γ-dependent mechanism, or through retrotranscytosis of secretory IgA-gliadin complexes by binding the transferrin receptor CD71 (37, 38). Once gluten peptides have reached the lamina propria, they are deamidated by the enzyme tissue transglutaminase, strongly enhancing epitope immunogenicity by increasing the affinity for HLA-DQ2 and DQ8 molecules expressed on the surface of antigen presenting cells, such as dendritic cells (39). These latter present deamidated gluten to gluten-reactive CD4^+^ T-cells that in turn induce a Th1- and Th17-mediated immune response, with an increased production of pro-inflammatory cytokines, especially IFN-γ (40, 41). Epithelium-derived thymic stromal lymphopoietin, that is a crucial cytokine for preserving immune tolerance, was found to be decreased in active CD, and this may explain the impaired differentiation of tolerogenic dendritic cells and the subsequent intestinal damage (42). Enterocyte apoptosis is driven by CD8^+^ intraepithelial lymphocytes (IELs) and sustained by the pro-inflammatory cytokine IL-15. This cytokine contributes to the inflammatory process in CD through different mechanisms, including the induction of the perforin-granzyme pathway, the increased IEL expression of natural killer receptors CD94 and NKG2D, and the abnormal production of IL-21 which in turns amplifies the whole damaging process (43, 44).

## Rationale for the use of epitope-based immunotherapy in celiac disease

The identification of gluten immunogenic peptides has a crucial importance in CD, either for the elucidation of immuno-pathogenic mechanisms responsible of gut damage, but above all for designing immunological therapies alternative to GFD. For long time, the characterization of pathogenic gluten epitope repertoire has been strongly hampered by the large heterogeneity of gluten proteins, and the limited amount of gut biopsy T-cells necessary for screening large peptide libraries (45, 46).

A step forward in the assessment of repertoire of gluten epitopes relevant for CD pathogenesis was given by the short oral gluten challenge procedure. Anderson et al. (47) established an innovative procedure that allows to detect in peripheral blood the gluten-specific T-cells of intestinal origin mobilized upon a short gluten consumption (3 days). A follow-up study by Tye-Din et al. (48) screened a large library of approximately 3000 gluten overlapping peptides for induction of IFN-γ responses in adult HLA-DQ2.5 CD patients undergoing a brief oral gluten challenge. Although, several peptides resulted to stimulate T-cells, only five epitopes (DQ2.5-glia-α1a, DQ2.5-glia-α2; DQ2.5-glia-ω-1, DQ2.5-glia-ω-2; DQ2.5-glia-γ-1) accounted for the great majority of T-cell stimulatory activity, due to a high cross reactivity rate. Gliadin peptide 33-mer, that is one of the most immunogenic fragments, includes DQ2.5-glia-α1a/b and DQ2.5-glia-α2 (49). On the contrary, peptide 31-43 (p31-43), that stimulates the synthesis and release of interleukin 15, is not immunogenic for T-cells, and therefore is not a suitable target of immunotherapy. A subsequent study from Hardy et al. (50) expanded such peptide repertoire analysis to a pediatric cohort of HLA-DQ2.5 CD volunteers. Of note, a comparable pattern of gluten peptide immunodominance between children and adults with CD was found. As the definition of gluten immunodominant peptides has allowed to develop a prototype of peptide-based therapeutic vaccine, the similarities in the repertoire of gluten peptides active in pediatric and adult CD patients will offer a great potentiality for a wide application of the peptide-based therapy for the treatment of CD.

## Clinical trial with Nexvax2

**Figure 1:**
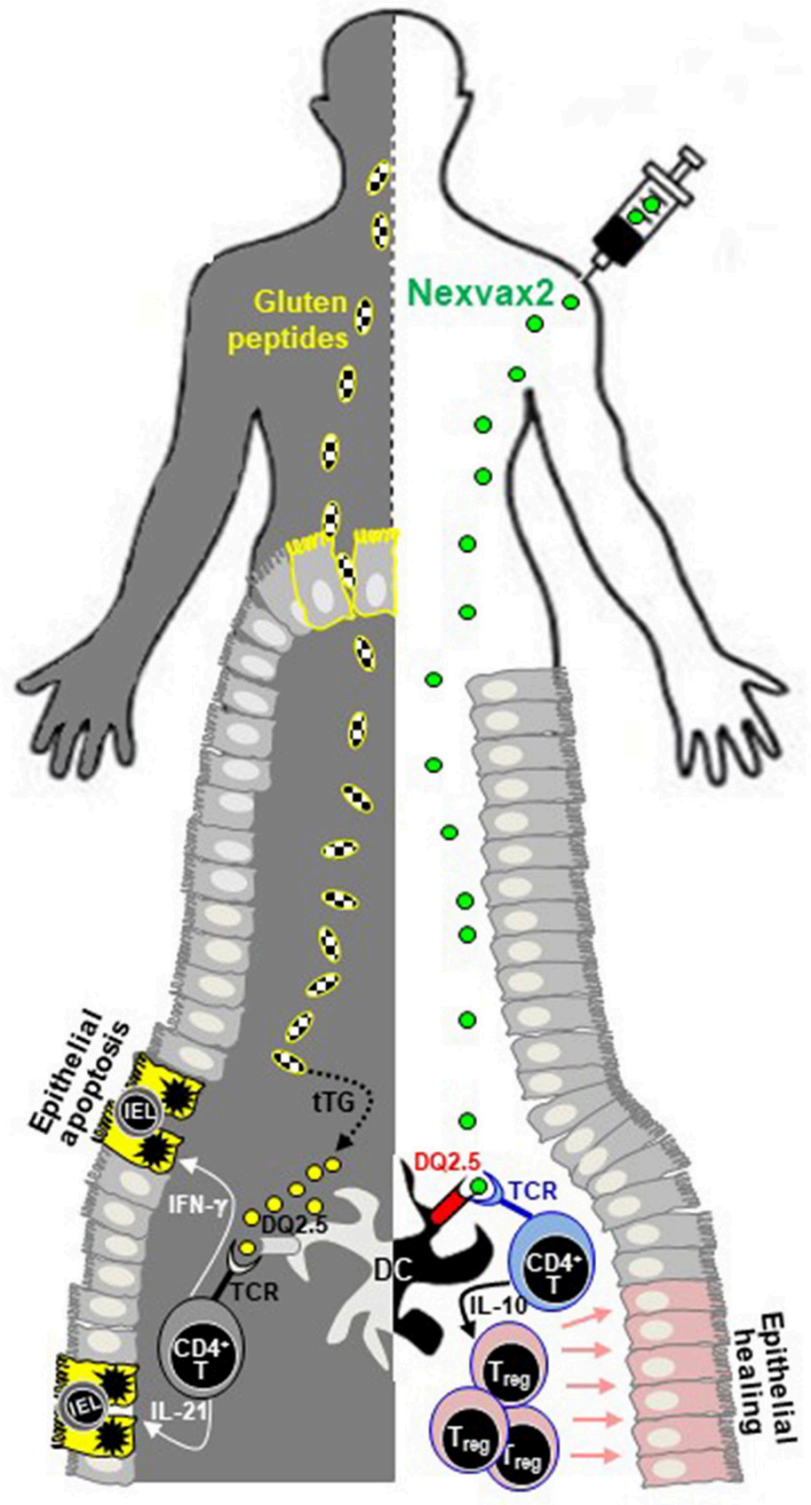
Schematic representation of Nexvax2 mechanism of action in patients with celiac disease. Nexvax2 is an epitope-specific vaccine that contains five different immunodominant gluten peptides. In patients suffering from celiac disease, the ingested gluten (left-hand side) is deamidated by tissue transglutaminase, a process that enhances its immunogenicity and eases recognition by HLA molecules expressed on dendritic cells. The subsequent increased production of pro-inflammatory cytokines by CD4^+^ T-cells is responsible for the small bowel mucosal damage, including villous atrophy and intraepithelial lymphocytosis. After Nexvax2 injection (right-hand side), peptides are recognized by HLA-DQ2.5, expressed on the surface of dendritic cells, and by the T-cell receptor of CD4^+^ T-cells, with subsequent increased production of interleukin-10. The repeated exposure to these peptides is thought to restore a normal tolerance toward gluten, thus healing the deranged small bowel mucosa of patients with celiac disease. DC, dendritic cell; IL, interleukin; IEL, intraepithelial lymphocyte; IFN, interferon; TCR, T-cell receptor; tTG, tissue transglutaminase.

Nexvax2 is an epitope-specific vaccine designed to treat CD, preventing the clinical and histological relapse after gluten consumption in patients with HLA-DQ2.5 genotype. It consists of three synthetic peptides of 15/16 amino acids that include five gluten peptides known to be most active in HLA-DQ2.5 CD patients, namely DQ2.5-glia-α1, DQ2.5-glia-α2 (both included in the 33-mer fragment), from α-gliadin, DQ2.5-glia-ω1, DQ2.5-glia-ω2, from ω-gliadin, and DQ2.5-hor3, from hordein proteins. The proof of concept of Nexvax2 is the inactivation of pathogenic T-cells reactive to the disease-relevant gluten peptides after intradermal administration (Figure 1). The intradermic injection of antigenic determinants has been demonstrated to efficiently tolerize the inflammatory T-cells specific for common allergens, in particular in individuals suffering from cat allergy (51, 52) Based on these encouraging results of ATI in cat allergy, the inventors of Nexvax2 vaccine have recently concluded a phase 1 double-blind, placebo-controlled, clinical study to evaluate safety, pharmacodynamics after repeated administrations, and mechanism of action of this drug (21). The study was conducted in different clinical centers in Australia, New Zealand, and the USA on adult HLA-DQ2.5^+^ celiac patients with disease remission and adherent to the GFD. One hundred and eight participants were randomly divided in two drug-experimental and placebo groups. The main inclusion criterium was the “transient” positive response to the short oral gluten challenge, in terms of mobilization in blood of gluten-reactive IFN-γ-secreting cells and Nexvax2 peptides. An adjuvant-free formulation of Nexvax2 was administered intradermally (supradeltoid region) and in ascending doses, precisely 60, 90, and 150 μg in the 3-doses branch (injected weekly over 15 days), and 150 and 300 μg in the 16-doses branch (injected twice weekly over 53 days).

The primary endpoint was the evaluation of adverse events during treatment, such as nausea, bloating, vomiting, and abdominal pain. Clinical symptoms were evaluated either by patient self-reported weekly Gastrointestinal Symptom Rating Scale or during periodic clinical visits. Gastrointestinal symptoms resembling gluten ingestion, such as nausea, vomiting, and headache, were reported at the time of the first injection and occurred in approximately 50% of treated patients within 2–5 h after the first dose. The great majority of patients complaining adverse effects were those receiving the 300 μg dose, and adverse effects were much less frequent at subsequent doses, thus indicating that a specific immune tolerance to Nexvax2 was reached during treatment. No serum conversion for CD-associated antibodies (anti-tissue transglutaminase, anti-gliadin deamidated peptides) was detected, as well as no antibodies to Nexvax2 were produced.

In the biopsy-cohorts, a gastroscopy was done between 15 and 28 days of treatment with Nexvax2 at 150 and 300 μg doses. No alteration of small intestinal morphology and lymphocyte density was reported, evaluated through villous/crypt length ratio and CD3+ IEL infiltration, respectively. Finally, patients who completed treatment at the maximal tolerated dose (150 μg) resulted non-responsive to a further oral gluten challenge as they had no IFN-γ production in response to Nexvax2. Notably, Nexvax2 treatment did not induce an unwanted immune activation in peripheral blood lymphocytes, as detected by IFN-γ ELISPOT assay.

## Perspective

The engineering of peptide-based immunotherapy that could specifically silence the adverse inflammatory cascade triggered by gluten proteins is currently one of the most promising therapies for CD. This type of immunotherapy aims at the complete recovery of immune tolerance to ingested gluten by targeting intestinal CD4+ T-cells that have a key pathogenic role in CD, without affecting the systemic immune responses. Such a strategy is particularly attractive as whole-protein allergen-based vaccines was demonstrated to be efficacious in allergic diseases in which causative antigenic peptides have been defined. The recent characterization of the repertoire of gluten epitopes responsible for mucosal inflammation in CD has allowed to design the Nexvax2 drug. Although the well-designed phase 1 trial by Goel et al. (21) has assessed the safety of Nexvax2, further studies are needed in order to demonstrate its efficacy to cure CD, i.e., to protect CD patients from the detrimental effects of gluten ingestion. Furthermore, it is of primary importance to assess which gluten dietary load the vaccine can protect from, as well as the duration of the effect. Second, the present vaccine is formulated with gluten T-cell epitope active in HLA-DQ2.5 patients only, thus excluding at least one-third of CD patients. Further efforts are required to optimize the epitope-specific immunotherapy aimed at allowing all CD patients to safely re-introduce gluten in their diet.

## Author contributions

All authors listed have contributed equally to this manuscript, have made a substantial, direct and intellectual contribution to the work, and approved it for publication.

### Conflict of interest statement

The authors declare that the research was conducted in the absence of any commercial or financial relationships that could be construed as a potential conflict of interest.

## Abbreviations

AIT: antigen-based immunotherapy
CD: celiac disease
GFD: gluten-free diet
HLA: human leukocyte antigen
IFN: interferon
IL: interleukin
IEL: intraepithelial lymphocyte.
